# Early onset pauciarticular arthritis is the major risk factor for naproxen-induced pseudoporphyria in juvenile idiopathic arthritis

**DOI:** 10.1186/ar2117

**Published:** 2007-01-31

**Authors:** Susanne G Schäd, Andrea Kraus, Imme Haubitz, Jiri Trcka, Henning Hamm, Hermann J Girschick

**Affiliations:** 1 Department of Dermatology, Venereology and Allergology, University of Würzburg, Josef-Schneider-Str, 97080 Würzburg, Germany; 2 Department of Dermatology and Venereology, University of Rostock, Augustenstr, 18055 Rostock, Germany; 3 Department of Pediatrics, Section of Pediatric Rheumatology and Osteology, University of Würzburg, Josef Schneider Str, 97080 Würzburg, Germany; 4 IT Centre, University of Würzburg, Am Hubland, 97074 Würzburg, Germany

## Abstract

Pseudoporphyria (PP) is characterized by skin fragility, blistering and scarring in sun-exposed skin areas without abnormalities in porphyrin metabolism. The phenylpropionic acid derivative group of nonsteroidal anti-inflammatory drugs, especially naproxen, is known to cause PP. Naproxen is currently one of the most prescribed drugs in the therapy of juvenile idiopathic arthritis (JIA). The prevalence of PP was determined in a 9-year retrospective study of children with JIA and associated diseases. In addition, we prospectively studied the incidence of PP in 196 patients (127 girls and 69 boys) with JIA and associated diseases treated with naproxen from July 2001 to March 2002. We compared these data with those from a matched control group with JIA and associated diseases not treated with naproxen in order to identify risk factors for development of PP. The incidence of PP in the group of children taking naproxen was 11.4%. PP was particularly frequent in children with the early-onset pauciarticular subtype of JIA (mean age 4.5 years). PP was associated with signs of disease activity, such as reduced haemoglobin (<11.75 g/dl), and increased leucocyte counts (>10,400/μl) and erythocyte sedimentation rate (>26 mm/hour). Comedications, especially chloroquine intake, appeared to be additional risk factors. The mean duration of naproxen therapy before the onset of PP was 18.1 months, and most children with PP developed their lesions within the first 2 years of naproxen treatment. JIA disease activity seems to be a confounding factor for PP. In particular, patients with early-onset pauciarticular JIA patients who have significant inflammation appear to be prone to developing PP upon treatment with naproxen.

## Introduction

Pseudoporphyria (PP) is a bullous disease of light-exposed skin with no abnormalities of porphyrin metabolism present [1]. Typical clinical manifestations are blistering, erosions, scarring and skin fragility, mimicking the photosensitivity reactions seen in erythropoietic porphyria and porphyria cutanea tarda (PCT) [2]. In contrast to these true porphyrias, in PP facial hypertrichosis, milia, hyperpigmentation and sclerodermoid skin changes are not observed. PP and PCT share the histological findings of subepidermal bullae, such as festooning of dermal papillae [3]. Immunofluorescence and ultrastructural features of lesional skin in PP also closely resemble those of PCT [4].

PP is associated with chronic renal failure with and without haemodialysis [5], exposure to UV light type A and use of tanning beds [6]. Numerous medications have been alleged to provoke PP, including nonsteroidal anti-inflammatory drugs (NSAIDs; especially propionic acid derivatives such as nabumetone, naproxen, oxaprozin and ketoprofen) [7-14], celecoxib [15], antibiotics (for instance, tetracycline and nalidixic acid) [16,17], diuretics (furosemide, triamterene with hydrochlorothiazide, chlortalidone and bumetanide) [18,19] and others (retinoids, cyclosporine, amiodarone, carisoprodol/acetylsalicyl acid, pyridoxine, flutamide, dapsone and oral contraceptive pills) [20-22].

PP is a considerable side effect of long-term treatment with NSAIDs, most commonly naproxen, in patients with juvenile idiopathic arthritis (JIA) [8,9,23,24]. Only a few studies of PP have been performed in children on long-term NSAID treatment. Thus far these studies have yielded a prevalence of PP between 10% and 12% in these patients, with fair skin and blue/grey eye colour established as risk factors [25]. Facial scarring improves slowly with time, but new skin lesions can appear even for weeks and months after discontinuation of treatment [9].

We conducted a retrospective study of children with JIA and associated diseases attending the paediatric rheumatology clinic of the University of Würzburg in order to determine the prevalence of naproxen-induced PP. Furthermore, we conducted a prospective cohort study of patients younger than 16 years with JIA and associated diseases treated with naproxen, and compared them with an age-matched control group not treated with naproxen in order to identify risk factors.

## Materials and methods

To determine the prevalence of naproxen-induced PP, we reviewed flowcharts and computerized data for all children (*n *= 395; age <16 years) with JIA and associated diseases who had been seen in the paediatric rheumatology clinic of the University of Würzburg between January 1993 and March 2002. In addition, for a parallel prospective study, all children with a history of naproxen-induced PP were compared with all children (*n *= 196) with JIA and associated diseases who presented at the clinic over a 9-month prospective period from July 2001 to March 2002. Informed consent was obtained from the parents. The study was performed in accordance with the principles of the revised declaration of Helsinki and has been approved by the ethics committee of the University of Würzburg.

All patients were examined for clinical manifestations of PP and underwent evaluation of immunological and laboratory parameters, including blood cell count with leucocytes, age-matched haemoglobin, platelets, erythrocyte sedimentation rate (ESR), liver function tests, serum immunoglobulins and antinuclear antibodies (ANAs). Parents and, if appropriate, patients were questioned regarding blistering, erosions, facial scarring, skin fragility, trauma, atopic and other skin diseases, sun exposure, skin care and skin phototypes (SPTs) I to VI, according to Fitzpatrick from Hamm and coworkers [26]. (Depending on the degree of tanning after UV light exposure, the following SPT categories are defined: SPT I, always burns, never tans; SPT II, usually burns, sometimes tans; SPT III, sometimes burns, usually tans; SPT IV, minimally burns, always tans; SPT V, rarely burns, tans profusely; and SPT VI, never burns, tans deeply.) Data including the medications used, dosages and comedications were reviewed and recorded. A questionnaire was sent to all parents with known naproxen-induced PP. Parents from 38 out of 45 children with PP filled out the questionnaire.

Photographs were taken of children with clinically suspected PP and reviewed by two paediatric rheumatologists and two independent dermatologists. Complete porphyrin testing of urine, stool and red blood cells was not done in all patients. However, other defined forms of porphyria, mainly erythropoietic porphyrias, PCT, and porphyria variegata and other blistering rashes in children (bullous polymorphic light eruption and autoimmune bullous diseases), were unlikely based on history, clinical features along with distribution of blisters, and course of disease in relation to intake of naproxen. Long-term information was obtained by review of follow-up visits and by standardized telephone interviews. Statistical analysis was done by χ^2 ^test and linear multiple discriminant analysis for categorial data and by *t*-tests for quantitative parameters.

## Results

### Incidence of pseudoporphyria

From January 1993 to March 2002, clinical symptoms of PP were observed in 45 out of 395 patients treated with naproxen. This represents a prevalence of naproxen-induced PP of 11.4%.

### Demographics and disease groups

A total of 196 children (128 girls and 68 boys) with JIA and associated diseases were included in the prospective study. The 45 patients with PP were compared with 96 patients treated with naproxen who did not develop PP and with 55 patients affected by JIA and associated diseases but who were not treated with naproxen (control group). In the control group, 41 children received an NSAID medication other than naproxen and 14 received no medication.

**Figure 1:**
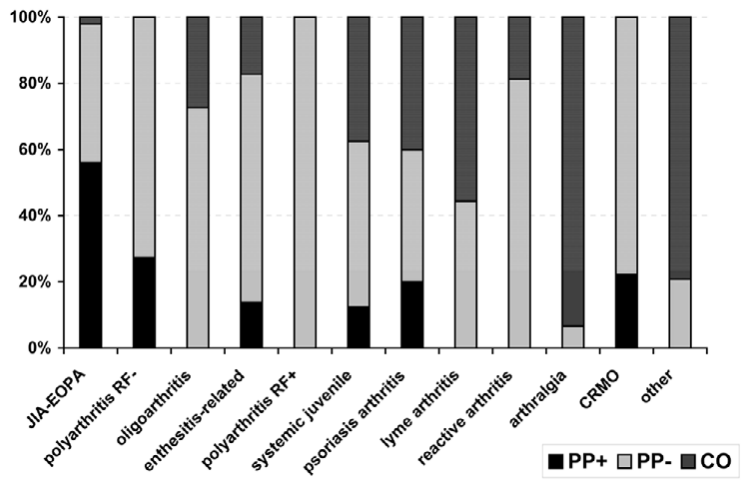
Juvenile idiopathic arthritis and related disorders in the different patient groups studied. CO, children not treated with naproxen (controls); CRMO, chronic recurrent multifocal osteomyelitis; JIA-EOPA: euvenile idiopathic arthritis, early-onset pauciarticular subtype; PP+, children treated with naproxen who developed pseudoporphyria; PP-, children treated with naproxen who did not develop pseudoporphyria.

Of the children affected by PP, 64% had the early-onset pauciarticular (EOPA) subtype of JIA, 16% had enthesitis-related arthritis, 9% had polyarthritis, 4% had chronic recurrent multifocal osteomyelitis, 2% had systemic juvenile arthritis, 2% had psoriasis arthritis and 2% had arthralgias (Figure 1). EOPA-JIA was significantly more common in the group with PP (64%) than in the group without PP (22%; *P *< 0.00001, χ^2 ^test) and in the control group (2%; *P *< 0.00001, χ^2 ^test). No significant difference was observed between sexes (with PP: 11 males and 34 females; without PP: 34 males and 62 females; controls: 23 males and 32 females). The mean age of onset of JIA in children with PP was 4.7 years (range 1.0 to 13.7 years), which was significantly younger than the mean age of 8.2 years in children without PP (range 0.3 to 16.8 years; *P *= 0.00001, Mann-Whitney U-test) and of 8.6 years in the control individuals (range 0.5 to 15.1 years; *P *= 0.00001, Mann-Whitney U-test; Table 1).

### Course of pseudoporphyria

**Figure 2:**
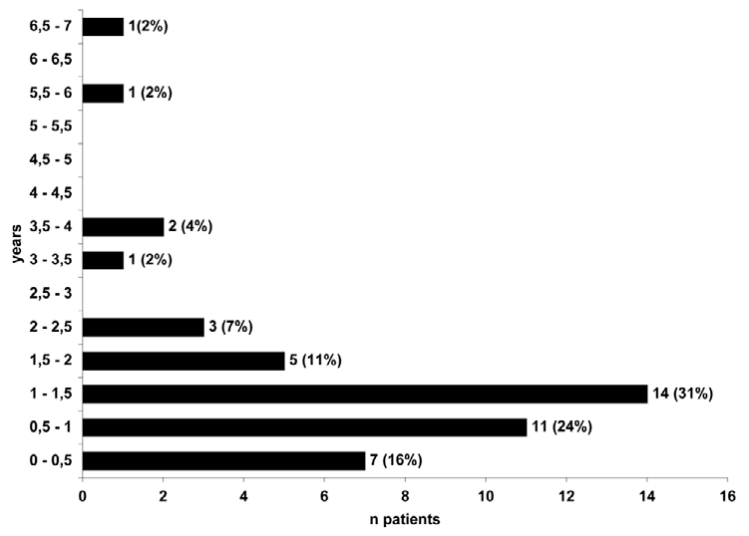
Duration (years) of treatment with naproxen before onset of pseudoporphyria. Note that 82% of children affected with pseudoporphyria developed skin lesions within 2 years of naproxen treatment.

The mean duration of naproxen therapy before onset of PP was 18.1 months (median 14 months; range 1–84 months). Of children with PP, 82% developed lesions within 2 years of naproxen treatment (Figure 2). There were no seasonal peaks in PP onset. No patient exhibited clinical features of facial hypertrichosis, miliae, hyperpigmentation, and sclerodermoid skin changes. A large proportion (93%) of skin lesions were facial. Lesions were observed most often on the cheeks (35%) and nose (33%), less commonly on the forehead (20%) and rarely on the chin (8%). The hand (2%), forearm (1%) and neck (1%) were very rarely affected.

**Figure 3:**
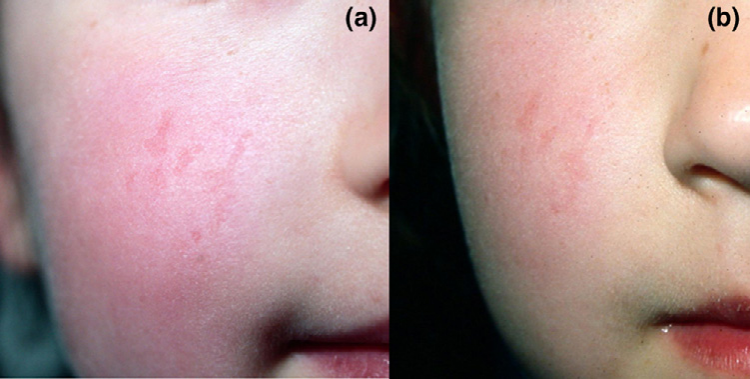
Clinical features of pseudoporphyria. **(a) **A few irregularly shaped, slightly depressed, erythematous scars on the right cheek of a 3-year-old girl with JIA-EOPA. These lesions appeared 14 months after the start of naproxen. **(b) **Nine months after discontinuation of naproxen the scars are still visible although slightly less depressed. JIA-EOPA: juvenile idiopathic arthritis, early-onset pauciarticular subtype.

Fifteen per cent of the children had a severe course of PP, with skin fragility and more than 10 skin lesions, including blistering and erosions followed by permanent scars. Forty-six per cent of children were moderately affected. Either skin fragility or 5–10 skin lesions occurred as blisters and erosions followed by persistent scars. Scars were often initially erythematous, then depressed, and had linear, angular, or bizarre outlines (Figure 3). Thirty-eight per cent of the children had a mild course of PP, with no skin fragility and only few (under five) skin lesions in terms of erythemas without scarring.

**Figure 4:**
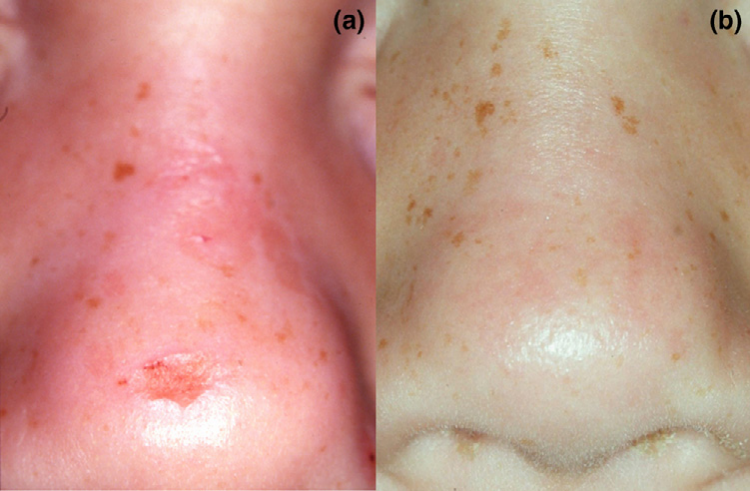
Clinical features of pseudoporphyria. **(a) **Crusted erosion on the tip of the nose of a 4-year-old boy with JIA-EOPA. The lesion occurred 6 months after the start of naproxen treatment. **(b) **Thirty-two months after discontinuation of naproxen the lesion appeared as irregular, light erythema. JIA-EOPA: juvenile idiopathic arthritis, early-onset pauciarticular subtype.

Complete resolution of the lesions after discontinuation of naproxen therapy was documented in 10 patients: five were free from disease within 6 months, two within 1 year, and one within each of 2, 3 and 4 years (Figure 4). Of children with PP, 21% continued to develop new skin lesions after discontinuation of naproxen therapy; clinical features continued to present in one patient for 1 month, in four patients for 2 months, and in three patients for 3 months after cessation of naproxen therapy. No first manifestation of PP was observed after cessation of naproxen treatment. PP did not occur during or after use of anti-inflammatory agents other than naproxen.

### Laboratory data

In the laboratory studies 61% of children affected by PP had a haemoglobin level below 11.8 g/dl (set as the lower cut off), but significantly fewer children without PP (34%; *P *= 0.0029, χ^2 ^test) and control children (24%; *P *= 0.00027, χ^2 ^test) had mild anaemia. The means of white blood cell counts were 10,440/μl in patients with PP, 8,000/μl in patients without PP, and 8,110/μl in the control children without naproxen intake. Thus, patients with PP had significantly higher white blood cell counts than did patients without PP (*P *= 0.00028, Mann-Whitney U-test) and the control children (*P *= 0.00018, Mann-Whitney U-test). Children with PP had a mean platelet count of 409,000/μl, children without PP had a platelet count of 362,000/μl, and the control group of 332,000/μl. These differences were statistically significant (Mann-Whitney U-test) between children with PP and control children (*P *= 0.0019) but not between children with PP and without PP (*P *= 0.37).

Seventy-one per cent of patients with PP, 51% of patients without PP and 30% of the control children had elevated (>11 mm/hour) ESR (mean 26 mm/hour, 22 mm/hour and 13 mm/hour, respectively). These differences were significant for the patients with PP in comparison with the patients without PP (*P *= 0.002, *t*-test for independent samples) and to the control group (*P *= 0.001, *t*-test for independent samples). Additionally, more children of the cohort affected with PP had a significantly higher ESR level than the cohort without PP and the control children (*P *= 0.027 and 0.001, respectively; χ^2 ^test). Comparing the mean ESR levels, a significant difference was seen between the children with and those without PP (*P *= 0.002, *t*-test for independent samples), as well as between the children with PP and the control group (*P *= 0.001, *t*-test for independent samples).

Abnormal levels of liver enzymes (lactate dehydrogenase, alanine aminotransferase, aspartate aminotransferase and γ-glutamyl transferase) were not observed in any group. There were no significant differences in mean immunoglobulin levels (IgG, IgA and IgM) between groups.

Of children with PP, 51% had detectable antinuclear antibodies (titre >1:80) in their serum. This proportion was significant higher than that in control children (24%; *P *= 0.012, χ^2 ^test), but not compared with children without PP (45%; *P *= 0.55, χ^2 ^test).

### Phototype and sun exposure

Fifty-eight per cent of children affected with PP had SPT I to II, which is a significantly greater proportion than that of children without PP (39%; *P *= 0.027, χ^2 ^test) but the difference compared with control children was not significant (53%; *P *= 0.45, χ^2 ^test). No significant differences between the groups were observed for the other SPTs, for the presence of freckles, for blue/grey eye colour, for fair hair colour, and for regular skin care. The distributions of SPT, blue/grey eye colour and fair hair colour did not differ significantly between JIA subtypes or associated diseases.

Patients were asked how long they had been exposed to sunlight during the summer and during the winter time. The intervals were under 3.5 hours/day and 3.5 hours/day or longer. For neither time frame could a significant difference be detected between the groups. In addition, 47% of patients with PP claimed to have undergone intense sun exposure during vacations before they developed PP; this proportion is significantly greater than that among control children (25% of the controls; *P *= 0.029, χ^2 ^test) but it was not significantly greater than that among patients without PP (31%; *P *= 0.082, χ^2 ^test). No significant differences could be detected between groups in daily sun exposure during the winter time, photoprotective measures and different sun protection factors used. In none of the groups was a correlation found between PP and atopic disease or psoriasis in the patients' personal or family histories.

### Duration and modes of therapy

The mean durations of treatment with naproxen for the cohorts with PP and without PP were 22.8 months and 20.2 months, respectively. The difference was not statistically significant (*P *= 0.12, Mann-Whitney U-test). Patients with PP and without PP were treated with mean dosages of naproxen of 16.7 and 15.5 mg/kg per day, respectively, and with maximal mean dosages of naproxen of 17.6 and 16.7 mg/kg per day, respectively. The mean daily dose of naproxen did not differ significantly between the two cohorts (*P *= 0.12, Mann-Whitney U-test). However, 39% of the patients taking a mean daily dose of naproxen above 14.0 mg/kg per day developed PP, which is significantly greater than the 4% among patients taking a mean daily dose of naproxen below 14.0 mg/kg per day (*P *= 0.000075, χ^2 ^test). During the course of disease the naproxen dosage had been increased in 42% of patients affected by PP as compared with 24% of patients who did not develop PP. This difference was statistically significant (*P *= 0.044, χ^2 ^test).

The population with PP had significantly more comedications (other NSAIDs, sulfasalazine, methotrexate, antibiotics, azathioprine, etanercept, cyclosporine, or chloroquine) than did the patients without PP (58% versus 38%; *P *= 0.024, χ^2 ^test). The only significant difference between the two groups for a certain drug was found for chloroquine; 27% of the children with PP were treated with chloroquine as compared with 6% of the children without PP (*P *= 0.0011, χ^2 ^test).

## Discussion

PP is a term used for PCT-like skin lesions arising in light-exposed areas but without underlying abnormalities in porphyrin metabolism. Identical to PCT, its features include UV light induced blisters and erosions, increased skin fragility and easy bruising followed by shallow scarring. NSAIDs, particularly naproxen, have been implicated in the pathogenesis of PP. Naproxen is routinely used in the therapy of children with JIA and associated diseases because of its good overall tolerability and its long half-life period.

In our retrospective review of 395 children with JIA and associated diseases attending the paediatric rheumatology clinic of the University of Würzburg, we found a prevalence of 11.4% (45/395) for naproxen-induced PP. This finding is in accordance with those of a few previous studies conducted in considerably smaller numbers of children. Lang and Finlayson [25] first reported a prevalence of 12% in their cohort of 74 naproxen-treated patients in Canada. De Silva and colleagues [10] found a similar prevalence of 10.9% in their group of 64 children with JIA in Scotland.

In our prospective cohort study we examined children and adolescents younger than 16 years with naproxen-treated JIA and associated diseases for skin signs and symptoms indicative of PP, and compared these patients with an age-matched control group not treated with naproxen. In the group of children affected by PP the EOPA subtype of JIA and young age were found to be risk factors for development of PP. ANA presence and sex seemed not to influence risk. In their smaller cohorts, de Silva and coworkers [10] and Lang and Finlayson [25] did not observe a significant difference in age at onset, subtype of JIA, presence of ANAs, or sex when they compared their PP patients with the remaining patients. In contrast, Levy and colleagues [27] detected increased ANA titres in 86% of children with PP. EOPA-JIA typically affects girls who are younger than 5 years old. An increased ANA titre is typical for this disease group.

Thus far, the mechanisms by which naproxen induces PP are not understood. Non-immune-mediated phototoxic reactions are mostly discussed. Because of its chemical structure, naproxen may absorb UV rays generating free oxygen radicals. This possibly results in cell membrane and tissue damage to the skin [28,29]. The long-lasting binding of naproxen to cell proteins might enhance this phototoxic reaction. In addition, the results of our study suggest that the EOPA subtype of JIA may be associated with increased skin photosensitivity. Further *in vitro *and controlled studies are necessary to elucidate the mechanisms by which naproxen induces PP and to improve management options for this clinical entity. Thus far, an animal model exists only for nalidixic acid-induced PP but not for NSAID [30].

PP occurred on average after 1.5 years treatment with naproxen and mainly within the first 2 years of treatment. Children and parents should be made aware of that fact. A severe course of PP was rarely observed; the skin was affected mildly to moderately in most cases (up to 10 skin lesions and skin fragility). The facial skin was involved most often.

At present, there are no clear guidelines on the management of naproxen-induced PP. With the first onset of PP, treatment with naproxen should be discontinued and substitution by another medication should be considered [31]. In our study, active PP lesions were still seen for up to 3 months after withdrawal of naproxen. Children and parents should be made aware of this also. Girschick and coworkers [9] observed ongoing skin fragility up to 6 months after cessation of naproxen intake. Furthermore, Levy and colleagues [27] and Allen and coworkers [32] suggested that the first manifestation of PP can occur after drug discontinuation. In our study, no first manifestations of PP were observed after termination of naproxen therapy. Special topical treatments or skin care do not seem necessary because most scars were no longer visible after 1 year. Only rarely did skin lesions persist for up to 4 years. Lang and Finlayson [25] reported fading but not complete disappearance of scars 1 year after the PP episode.

In our laboratory studies (Table 1), anaemia (haemoglobin <11.8 g/dl), increased white blood cell count and elevated ESR were found to be further risk factors for development of naproxen-induced PP. Thus far, no other authors have investigated the association of these laboratory data with development of PP. Reduced haemoglobin in blood results in reduced haemoglobin in skin, and these fewer oxygen carriers are therefore highly saturated with oxygen. Hence, oxygen may be released more easily and may lead to enhanced reactions in the skin in the presence of phototoxic metabolites. PP has also been observed in patients with chronic renal failure. These patients generally suffer from renal anaemia. However, the laboratory data indicating reduced haemoglobin, increased white blood cell count and elevated ESR together suggest increased disease activity of PP-affected patients in JIA and associated diseases. Even though a confounder effect in this regard cannot be excluded, disease activity (as defined by the laboratory parameters haemoglobin, leucocytes and ESR) *per se *seems to be of relevance to the development of PP, because the naproxen dosage did not differ significantly between the two cohorts of children with and without PP. High levels of inflammatory parameters appear to favour the phototoxic reaction of naproxen in the skin. In the present study, no further laboratory data were found to be risk factors.

Our study cannot confirm the observations of de Silva and coworkers [10] and Wallace and colleagues [33] that blue/gray eye colour represents a risk factor for development of PP. However, in accordance with the latter group, we also found that children with fair skin (SPT I to II) were at greater risk for developing PP. The presence of freckles did not influence the risk for development of PP in the present study.

Surprisingly, photoprotective measures such as application of sunscreens (sun protection factor >20) did not appear to prevent the development of skin fragility or skin lesions. Adequate photoprotection might not have been achieved, and the use of sunscreens with higher sun protection factors would perhaps have been more efficient. Half of the children with PP had intense sun exposure during vacation shortly before developing PP. Otherwise, the amount of daily sun exposure during summer or winter time did not appear to influence the risk for development for PP. Correspondingly, de Silva and coworkers [10] found no significant difference in the amount of sun exposure or prior history of sunburn between children affected by PP and those in control groups. In our study, PP occurred in summer and winter months at equal frequencies, without any seasonal peak. Because the winter time is associated with less intense UV radiation, the intensity of UV radiation appears not to have an important influence on the development of PP. However, because PP lesions only occurred in sun-exposed skin (mostly on the face), and children with SPT I to II were at greater risk for developing PP, UV radiation still appears to have some influence on the development of PP, even though no seasonal peak of PP was observed. Considering the lengthy duration of naproxen treatment until PP occurred (18.1 months of therapy), one could argue that accumulation of UV radiation over a particular threshold might be the triggering factor, making any seasonal influence less overt. Therefore, in our opinion, naproxen-treated patients should be informed about sun protection and adivsed on thorough application of potent sunscreens (sun protection factor >30). Regular skin care and application of emollients did not prevent the development of PP. A history of atopic disease or psoriasis also did not appear to influence PP risk.

Children affected by PP received naproxen for a similar length of time and at a similar dosage as did naproxen-treated children who did not develop PP. Duration of treatment and mean daily dosage of naproxen were therefore not found to be risk factors for developing PP. This finding is in accordance with observations reported by de Silva and coworkers [10]. However, the increase in naproxen dosage over the course of disease and comedications, especially chloroquine, both appear to increase PP risk. With increased naproxen dosage, a greater amount of naproxen and its phototoxic metabolites might accumulate in the skin, which presumably may enhance phototoxic reactions. This could also be triggered by interactions with comedications. In addition, an increase in naproxen dosage and use of comedications are mostly a consequence of increased disease activity. As indicated in the present study JIA disease activity, as indicated by laboratory parameters of inflammation (hemoglobina, leucocyte count and ESR), is an important risk factor for development of PP. Among the comedications used, chloroquine is associated with increased PP risk. Chloroquine itself is reported to induce skin erythema, hyperpigmentation and photodynamic reactions [34]. Like naproxen, chloroquine also has a long half-life (40 days).

Even though the study presented is not based on blinded generation of data, we based the diagnosis of PP on clinical examination and additional evaluation of photographs by independent dermatologists. Nevertheless, we cannot completely exclude the possibility that the paediatric rheumatologist was biased, based on previous experience that the EOPA JIA subgroup appears to be prone to developing PP. In this regard, the true risk factors for PP appear to be SPT I to II, because children affected by EOPA-JIA in our cohort were predominantly of Scandinavian ancestry, and so fair skin is more likely to be present in these children. In addition, they are generally young, making early age another confounder.

Further studies to investigate the mechanisms of naproxen-induced PP, particularly in the EOPA-JIA subtype, could be helpful in developing further criteria for treatment with naproxen.

## Conclusion

We found a prevalence of PP among children with JIA treated with naproxen of 11.4%. JIA disease activity, as indicated by low haemoglobin level, increased white blood cell count and elevated ESR, is an important risk factor for development of PP. The potential risks associated with naproxen treatment should be carefully considered, especially in fair-skinned children, who are young (<4.5 years) and diagnosed with EOPA-JIA. Particular caution is warranted within the first 2 years of naproxen intake and in the case of comedication with chloroquine.

## Abbreviations

ANA = antinuclear antibody; EOPA = early onset pauciarticular; ESR = erythrocyte sedimentation rate; JIA = juvenile idiopathic arthritis; NSAID = nonsteroidal anti-inflammatory drug; PCT = porphyria cutanea tarda; PP = pseudoporphyria; SPT = skin phototype.

## Competing interests

The authors declare that they have no competing interests.

## Authors' contributions

SGS, AK, JT, HH, and HJG contributed to the clinical care of patients; IH was responsible for statistical analysis. HJG led the pediatric clinical care and treatment strategies. SGS, HH, and JT conducted dermatologic diagnosis and care. All authors read and approved the final manuscript.

## Figures and Tables

**Table 1:** Characteristics of the different patient groups studied with juvenile idiopathic arthritis and related disorders

Characteristics	PP+ (*n *= 45)	PP- (*n *= 96)	CO (*n *= 55)	PP+ versus PP-	PP+ versus CO
Female (%)	76	65	58		
Mean age (years)	4.7	8.2	8.6	*P *< 0.01^a^	*P *< 0.01^a^
Laboratory findings					
Haemoglobin level <11.8 g/dl (%)	61	34	24	*P *< 0.01^b^	*P *< 0.01^b^
Mean white blood cell count (cells/μl)	10,440	8,000	8,110	*P *< 0.01^a^	*P *< 0.01^a^
Mean platelet count (×10e^3^/μl)	409	362	332		*P *< 0.01^a^
ESR >11 mm/hour (%)	71	51	30	*P *< 0.01^c^	*P *< 0.01^c^
Positivity for ANAs (%)	51	45	24		*P *< 0.05^b^
Factors relating to the skin					
SPT I and II (%)	58	39	53	*P *< 0.05^b^	
Freckles (%)	42	34	36		
Blue/grey eye colour (%)	68	72	60		
Fair hair colour (%)	61	50	51		
Regular skin care (%)	82	75	82		
Intense sun exposure during vacation (%)	47	31	25		*P *< 0.05^b^
Photoprotective measures (%)	89	83	85		
Application of sun screen with >20 SPF (%)	44	50	42		
Atopic diathesis (%)	21	25	16		
Psoriasis (%)	8	3	2		
Factors relating to naproxen therapy and comedication					
Mean duration of naproxen therapy (months)	22.8	20.2	-		
Mean dosage of naproxen (mg/kg per day)	16.7	15.5	-		
Maximal mean dosage of naproxen (mg/kg per day)	17.6	16.7	-		
Increased naproxen dosage during the course of treatment (%)	42	24	-	*P *< 0.05^b^	
Comedication (%)	58	38	-	*P *< 0.05^b^	
Chloroquine as comedication (%)	27	6	-	*P *< 0.01^b^	

^a^Mann-Whitney U-test. ^b^χ^2 ^test. ^c^*t*-test for independent samples. ANA, antinuclear antibody; CO, children not treated with naproxen (controls); ESR, erythrocyte sedimentation rate; PP+, children treated with naproxen who developed pseudoporphyria; PP-, children treated with naproxen who did not develop pseudoporphyria; SPF, sun protection factor; SPT, skin phototype.
